# Inhibitory effects of resistant starch (RS3) as a carrier for stachyose on dextran sulfate sodium-induced ulcerative colitis in C57BL/6 mice

**DOI:** 10.3892/etm.2013.1280

**Published:** 2013-09-02

**Authors:** YU QIAN, XIN ZHAO, JIA-LE SONG, KAI ZHU, PENG SUN, GUI-JIE LI, RUI WANG, JIAN-QUAN KAN

**Affiliations:** 1 College of Food Science, Southwest University, Chongqing 400715;; 2 Department of Biological and Chemical Engineering, Chongqing University of Education, Na’an, Chongqing 400067, P.R. China;; 3 Department of Food Science and Nutrition, Pusan National University, Busan 609-735, Republic of Korea

**Keywords:** resistant starch, C57BL/6 mice, cytokine, stachyose

## Abstract

The aim of this study was to determine the effects of resistant starch 3 (RS3) as a carrier for stachyose on dextran sulfate sodium (DSS)-induced colitis in C57BL/6 mice. RS3 microspheres carrying stachyose (RS3 + stachyose) were produced and evaluated as a potentially improved colitis therapy for this study. The body weights of the mice treated with RS3 + stachyose were higher compared with those of DSS-treated control mice. RS3 + stachyose reduced the levels of the serum pro-inflammatory cytokines IL-6 and TNF-α to a greater extent compared with the same concentration of stachyose combined with ordinary starch (stachyose + starch). Histopathological examination of sections of colon tissues showed that the RS3 + stachyose group recovered well from colitis; however, the tissue sections of the stachyose + starch group presented necrosis to a more serious degree. These results suggest that stachyose with an RS3 carrier has better preventative effects on colitis than stachyose alone in mice.

## Introduction

Resistant starch (RS) is the sum of starch and starch degradation products that are not absorbed in the small intestine since they are resistant to enzymatic digestion (1). RS3 is retrograded starch, formed on cooling in processed foods, including cooled cooked potato, bread and cornflakes (2). RS appears to have a number of physiological effects, including weight control, prevention of diabetes, lipid reduction and promotion of inorganic salt absorption (3). Stachyose is a tetrasaccharide present in the tubers of the Chinese artichoke (4). It is not decomposed by digestive enzymes and it will be changed in specific conditions when in direct contact with the intestinal tract (5). It increases probiotic activity; intestinal flora become contained within the intestines, form a thick protective film against bacteria and stop the proliferation of toxins within the intestinal tract (6). Stachyose also promotes intestinal peristalsis and accelerates the excretion of pathogens and toxins (7).

Ulcerative colitis is a type of inflammatory bowel disease that affects the lining of the large intestine and rectum (8). The symptoms vary in severity and may start slowly or suddenly. Approximately half of patients have only mild symptoms. Others have more severe attacks that occur more frequently. A number of factors lead to attacks, including respiratory infections or physical stress. Ulcerative colitis is one of the two main forms of chronic inflammatory disease of the gastrointestinal tract (9); the other form is Crohn’s disease. Normally, the large intestine absorbs water from stools and changes them from a liquid to a solid. In ulcerative colitis, inflammation causes loss of the lining of the colon, leading to bleeding, diarrhea and abdominal discomfort (10). Cytokines may be centralized around this organ as it hosts cells that are highly susceptible to the action of these proteins. Cytokines, such as IL-6 and TNF-α, are small proteins that are produced and released from a number of cells under physiological and pathological conditions (11). IL-6 is increasingly recognized as an almost ubiquitous participant in numerous types of inflammatory processes (12). TNF-α is a macrophage-derived cytokine with chemotactic potency, which has been implicated in the acute phase reaction under various inflammatory conditions (13).

In the present study, RS3 was used as a carrier for stachyose and its preventative effect on colitis was examined. The levels of the inflammation-related cytokines IL-6 and TNF-α were used to determine the preventative effects on dextran sulfate sodium (DSS)-induced colitis in mice. Colon tissue histology was also used to determine the preventative effects *in vivo.*

## Materials and methods

### Chemicals

RS3 was supplied by National Starch (Sterling Forest, NY, USA). RS3 (7 g) was added to 65 ml water, 0.4 g emulsifier (sucrose esters of fatty acids, Liuzhou Gaotong Food Chemicals Co., Ltd., Liuzhou, China), 100 ml soybean oil, 1.2 g crosslinking agent (POCl_3_) and 0.6 g initiator (cerium ammonium nitrate) at pH 8–9, 55°C. The products were left to crosslink for 3 h, thereby producing the RS3 microspheres. Then, 0.05 g stachyose (Sigma, St Louis, MO, USA) was added at 37°C for 3 h static adsorption. Ethanol was used to wash and filter the stachyose RS3 microspheres. Ordinary starch (COFCO Corporation Chongqing Company, Chongqing, China) was transformed into stachyose-containing starch using the same method. Mouse diets with a 15% starch content were prepared from the two kinds of stachyose.

### Animals

Female C57BL/6 mice (n=40, 7 weeks old) were purchased from the Experimental Animal Center of Chongqing Medical University (Chongqing, China). The mice were maintained in a temperature-controlled (temperature, 25±2°C; relative humidity, 50±5%) facility with a 12-h light/dark cycle and free access to a standard mouse chow diet and water.

### DSS-induced colitis model

The mice were divided into four groups (n=10 each). The normal group received a standard diet and water during the experimental period. The mice in the RS3 + stachyose and starch + stachyose groups were fed with a mouse diet containing 15% stachyose-containing RS3 and starch microspheres, respectively, for 2 weeks. In the second week, ulcerative colitis was induced in the control and sample groups by providing water containing 5% (wt/wt) DSS (molecular weight, 36,000–50,000; MP Biomedicals, Solon, OH, USA) *ad libitum* for 7 days, as described previously (14). The body weight was recorded daily and the colon length was measured. These experiments followed a protocol approved by the Animal Ethics Committee of Chongqing Medical University (Chongqing, China).

### Analysis of inflammation-related cytokines in serum by enzyme-linked immunosorbent assay (ELISA)

For the serum cytokine assay, blood from the inferior vena cava was collected in a tube and centrifuged at 1,100 × g, 4°C for 10 min. The serum was aspirated and assayed as described below. Concentrations of inflammatory-related cytokines IL-6 and TNF-α in serum were measured by ELISA according to the manufacturer’s instructions (Biolegend, San Diego, CA, USA). Briefly, biotinylated antibody reagent was added to 96-well plates, then supernatants of homogenized serum were added and the plates were incubated at 37°C in CO_2_ for 2 h. After washing with PBS, streptavidin-horseradish peroxidase (HRP) solution was added and the plate was incubated for 30 min at room temperature. The absorbance was measured at 450 nm using a microplate reader (iMark; Bio-Rad, Hercules, CA, USA) (15).

### Analysis of serum levels of superoxide dismutase (SOD)

For the blood biochemical assay, blood from the inferior vena cava was collected in a tube and centrifuged at 3,000 rpm, 4°C for 10 min. The serum levels of SOD were determined using a superoxide dismutase assay kit (Asan Pharm. Co. Ltd., Seoul, South Korea). The kit contained all reagents required for determining SOD activity in an indirect assay method based on xanthine oxidase (XO) and a color reagent that provides linearity of test results over a broad range. Briefly, the biotin-antibody reagent was added to 96-well plates, the supernatants of homogenized colon tissue were added and the plates were incubated at 37°C in CO_2_ for 1 h. After each well was aspirated and washed, HRP-avidin reagent was added to each well and the plates were incubated for 1 h at 37°C. The absorbance was measured at 450 nm using a microplate reader.

### Histological examination

At the end of the experimental period, the colon tissues were removed and cleaned in saline to remove fecal residue. The colon tissues were fixed in 10% (v/v) buffered formalin and embedded in paraffin. Then, 4-*μ*m-thick slices from paraffin sections were stained with hematoxylin and eosin (H&E) prior to microscopic observation. Histological analysis was conducted by a pathologist who was unaware of the experimental protocol.

### Statistical analysis

Data are presented as mean ± standard deviation (SD). Differences between the mean values for individual groups were assessed with one-way analysis of variance (ANOVA) with Duncan’s multiple range test. P<0.05 was considered to indicate a statistically significant differences. SAS version 9.1 (SAS Institute Inc., Cary, NC, USA) was used for the statistical analyses.

## Results

### Changes in body weight

**Figure 1.**
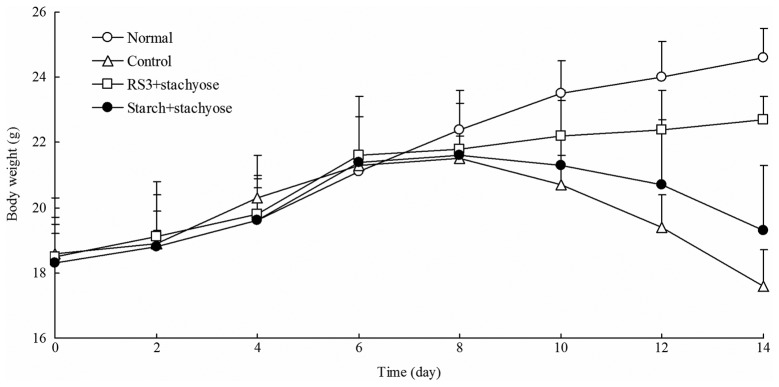
Effect of RS3 + stachyose on the body weights of C57BL/6 mice with DSS-induced colitis. RS3, resistant starch 3; DSS, dextran sulfate sodium.

The normal mice had a normal diet and their body weights increased in the whole process of experiment. The body weights of the control mice with DSS-induced colitis were significantly decreased after 7 days. As shown in Fig. 1, following the initiation of DSS-induced colitis, the body weights of the mice in the RS3 + stachyose and starch + stachyose groups were significantly lower compared with those of the normal mice. The mice in the RS3 +stachyose group had higher body weights than the mice in the starch + stachyose group (P<0.05). After 7 days, the body weight of RS3 + stachyose group and starch + stachyose group mice were higher than that of control group mice, and the body weight of the RS3 + stachyose group was higher than starch + stachyose group.

### Changes in colon length

**Figure 2.**
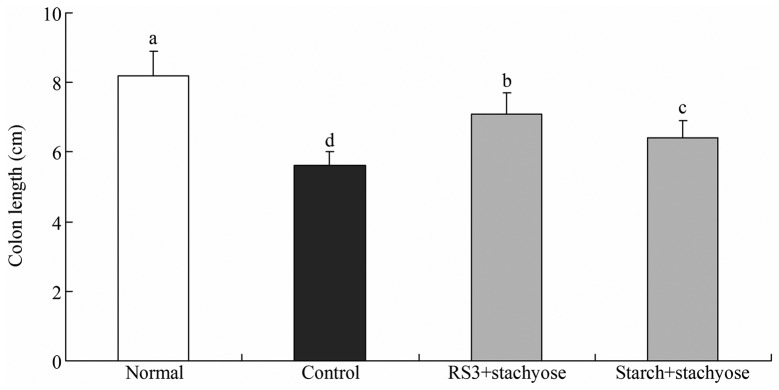
Effect of RS3 + stachyose on colon length in C57BL/6 mice with DSS-induced colitis. a-d, mean values with different letters over the bars are significantly different (P<0.05) according to Duncan’s multiple range test. RS, resistant starch; DSS, dextran sulfate sodium.

The total colonic length was significantly shorter in the DSS-treated mice (control group and sample treated group) compared with the normal mice as shown in Fig. 2 (P<0.05). The normal mice had the longest colon length at 8.2±0.7 cm and the colon length of control mice was the shortest (5.6±0.4 cm). The total colonic length was longer in the RS3 + stachyose-treated mice (7.1±0.6 cm) than in the starch + stachyose-treated mice (6.4±0.5 cm). The significant shortening of the colonic length in DSS-treated mice indicates that DSS contributed to the process of edematous changes in the colon in DSS-induced colitis.

### Effect of RS3 + stachyose on serum levels of IL-6 and TNF-α

**Figure 3.**
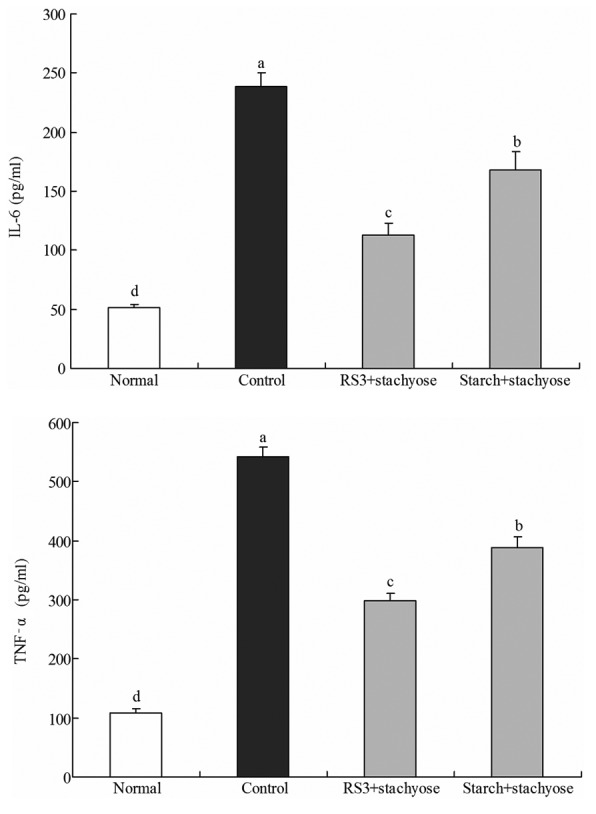
Serum IL-6 and TNF-α levels of mice with DSS-induced colitis treated with RS3 + stachyose. a-d, mean values with different letters over the bars are significantly different (P<0.05) according to Duncan’s multiple range test. RS, resistant starch; DSS, dextran sulfate sodium.

The IL-6 level of normal mice was 51.1±2.7 pg/ml; however, in control mice the IL-6 level was significantly increased to 238.6±11.6 pg/ml. The levels of IL-6 in mice fed with RS3 + stachyose and starch + stachyose were 112.6±10.6 and 168.4±15.4 pg/ml, respectively (Fig. 3). The TNF-α levels in the normal, control, RS3 + stachyose-treated and starch + stachyose-treated mice were 108.6±7.8, 542.3±16.3, 298.4±12.5 and 388.3±17.7 pg/ml, respectively. The serum IL-6 and TNF-α levels in the mice in the RS3 + stachyose-treated groups were significantly lower compared with those in the control and starch + stachyose groups.

### Effect of RS3+stachyose on serum levels of SOD

**Figure 4.**
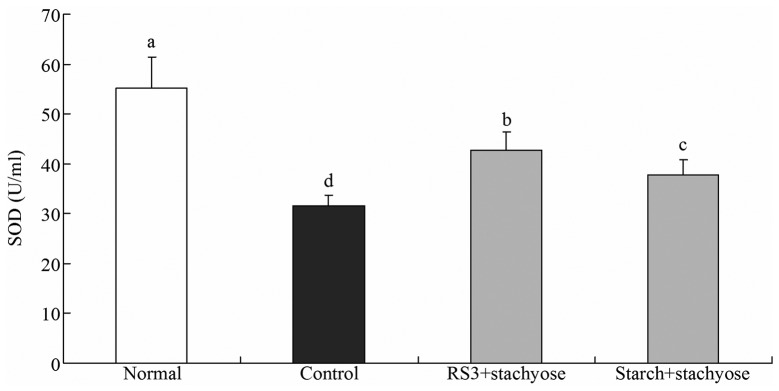
Serum SOD levels of mice with DSS-induced colitis treated with RS3 + stachyose. a-d, mean values with different letters over the bars are significantly different (P<0.05) according to Duncan’s multiple range test. SOD, superoxide dismutase; RS, resistant starch; DSS, dextran sulfate sodium.

The SOD level of normal mice was 55.2±6.3 U/ml and control mice demonstrated the lowest level at 31.6±2.1 U/ml. The SOD levels in the RS3 + stachyose- and starch + stachyose-treated mice increased to 42.8±3.6 and 37.7±3.2 U/ml, respectively (Fig. 4).

### Effect of RS3 + stachyose on histological damage in the colon tissue of mice with DSS-induced colitis

**Figure 5.**
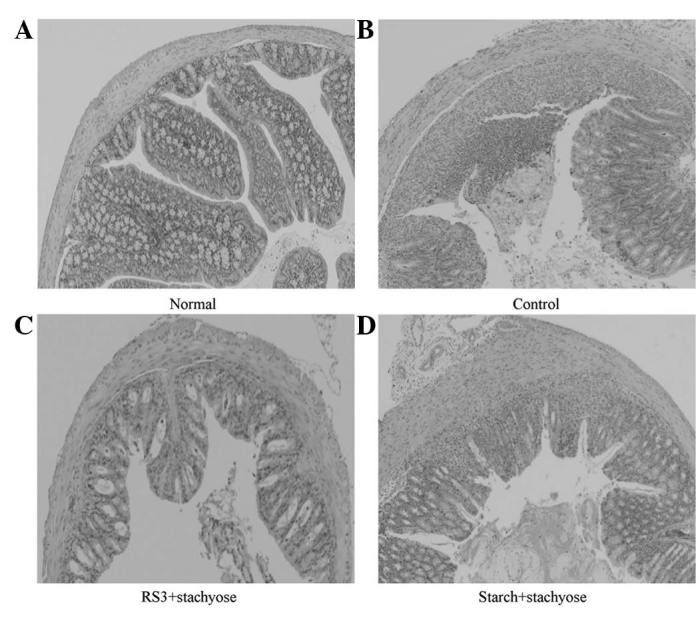
Microscopic observations of the effects of RS3 + stachyose on histological damage in the colon tissues of mice with DSS-induced colitis (magnification, ×40). RS, resistant starch; DSS, dextran sulfate sodium.

After collecting colonic tissue samples, the severity of colitis was characterized by macroscopic examination of the colon and histological analysis of H&E stained colonic sections. The inhibitory effects of the stachyose-containing microspheres on the DSS-induced damage of the colon tissue are shown in Fig. 5. H&E staining analysis indicated that the administration of DSS markedly increased the severity of the colitis compared with that in the normal mice (Fig. 5B). A typical lesion of the colon in the DSS-treated group manifested multifocal areas, mucosal erosion, loss of epithelial and goblet cells, shortening and collapse of crypts, and submucosal edema. RS3 + stachyose and starch + stachyose administration reduced the lesions of the colon in DSS-induced colitis in the starch carrier technique-dependent manner, as shown in Fig. 5C and D. The protective and healing effects of RS3 + stachyose on colon damage were more prominent (Fig. 5C).

## Discussion

The most significant symptom in DSS-induced colitis in mice is body weight loss (16). Colon length may be measured to determine the severity of colitis. Changes in colon weight and colon length reflect the inflammatory status in mice with DSS-induced colitis and also demonstrate which treatment has preventative effects against DSS-induced colitis in mice (17).

The serum IL-6 and TNF-α levels in patients with inflammatory diseases are higher compared with those in healthy individuals (18). Lower levels of IL-6 and TNF-α are indicative of improved anti-inflammatory effects. IL-6 acts as a pro-inflammatory and anti-inflammatory cytokine. IL-6 is secreted by T cells and macrophages to stimulate the immune response, particularly in tissue damage leading to inflammation. IL-6 also plays a role in fighting infection (19). TNF-α is a cytokine involved in systemic inflammation and is a member of a group of cytokines that stimulate the acute phase reaction (20). IL-6 and TNF-α are key mediators in a number of experimental colitis models (21).

SOD is a protein that neutralizes free radicals. This protein is an enzyme that inhibits or regulates a specific chemical or signaling action (22); specifically, it is one of the important anti-oxidative enzymes. It catalyzes the dismutation of the superoxide anion into hydrogen peroxide and molecular oxygen (23). The rate of the reduction of a superoxide anion is linearly related to the XO activity and is inhibited by SOD. Therefore, the inhibitory activity of SOD may be determined by a colorimetric method. In normal body cells, SOD protects the cytoplasmic structures from damage caused by free radicals (24).

RS is not digested in the stomach. It resists enzymatic decomposition and enters into the colon as a nutrient source for colonic bacteria. Through fermentation, these microbes metabolize carbohydrates and generate short-chain fatty acids, including butyric acid. Butyric acid lowers the pH value of the colon and feces to promote colon health and prevent colitis (25).

Under the activation of stachyose, bacteria produce a large amount of short-chain fatty acids, which reduce intestinal pH and Eh values, prevent the growth of harmful bacteria and promote intestinal peristalsis in order to accelerate the excretion of pathogenic bacteria and toxins. Stachyose itself decomposes immunity factors, including manninotriose and melibiose. These factors induce the body to produce more endocrine immunoglobulin (IgA) and thereby neutralize bioactive antigens such as viruses, toxins and pathogenic bacteria. These factors have an extensive protective function (26) and improve immunity. Stachyose has a positive effect on reducing the intestinal pH value, adjusting the intestinal micro-ecological balance, inhibiting the metabolism of pathogenic and spoilage organisms, promoting intestinal peristalsis and immunity, and curing enteritis (5).

In the present study, mice consumed a mouse diet that included RS3 and stachyose. Stachyose combined with RS3 is not absorbed in the stomach. After it enters into the colon, RS3 is broken down by the intestinal bacteria and stachyose is released. This is advantageous to the proliferation of intestinal probiotics and has the effect of preventing colitis. By contrast, large amounts of ordinary starch are decomposed by enzymes prior to entering the colon; therefore, stachyose is only partly absorbed in the colon, which reduces its efficacy. As indicated by the experimental results, combining RS3 with stachyose enables the full efficacy of stachyose to be utilized. Compared with the direct use of stachyose, this method produces better preventative effects against colitis.
